# CSF1R mutations in an Italian population of early-onset dementia: a case series

**DOI:** 10.1007/s00415-026-13643-1

**Published:** 2026-02-04

**Authors:** Beatrice Pancaldi, Andrea Mastrangelo, Alessandro Zilioli, Edoardo Ruggeri, Veria Vacchiano, Elena Pasini, Gabriele Busi, Piero Parchi, Marco Spallazzi, Sabina Capellari

**Affiliations:** 1 Department of Medicine and Surgery, Unit of Neurology, University of Parma, Parma, Italy; 2 IRCCS Istituto Delle Scienze Neurologiche Di Bologna, Bologna, Italy; 3 Department of Biomedical and Neuromotor Sciences, University of Bologna, Via Altura 3, 40139 Bologna, Italy; 4 Department of Medicine and Surgery, Unit of Neurology, University-Hospital of Parma, Parma, Italy

**Keywords:** Dementia, CSF1R, White matter, Genetics, Cerebrospinal fluid, Leukodystrophy

## Abstract

The diagnostic approach to subjects with early-onset dementia (EOD) is often challenging due to the broader range of possible etiologies as compared to late-onset dementia cases. Pathogenic variants in *CSF1R* gene have been increasingly reported in subjects with EOD, mostly clinically mimicking behavioral variant of frontotemporal dementia (bvFTD). Here we screened for variants in *CSF1R* gene in a large cohort of dementia patients consecutively referred for genetic analysis to an Italian tertiary center between 2005 and 2024 (*n* = 2163). Sequence of *CSF1R* gene was determined with next-generation sequencing through either a dedicated panel or whole-exome sequencing. Pathogenic variants or variants of uncertain significance with higher evidence of pathogenicity were found in four participants (one female); three of these were not previously reported. Clinical data were collected, including brain magnetic resonance imaging and neuropsychological assessment. Cerebrospinal fluid (CSF) levels of neurofilament light chain (NfL) protein were measured. A family history of dementia was present in one subject. Mean age at onset was 51.5. Seizures were the presenting symptom in two cases and later appeared in other two. Two subjects presented with behavioral disturbances, resembling early bvFTD. Neuropsychological assessment revealed executive and language impairment in most cases. Anterior-predominant atrophy, symmetric white-matter involvement, and serpentine calcifications were the most common imaging abnormalities. High CSF NfL levels were found in all cases, with two of them showing markedly elevated values. CSF1R-related disease should be considered in EOD subjects, especially those presenting with executive/language deficits, seizures, white matter involvement, and markedly elevated CSF NfL levels.

**Supplementary Information:**

The online version contains supplementary material available at 10.1007/s00415-026-13643-1.

## Introduction

Early-onset dementia (EOD) is a condition characterized by progressive cognitive decline that affects daily life and work functioning with onset before the age of 65 [1]. It encompasses a heterogeneous spectrum of primary and secondary dementias, representing a significant challenge for clinicians [2].

EOD clinical features are more variable than those typically observed in late-onset cases. In younger individuals, early symptoms often include changes in behavior, language, personality, and executive function [3].

Early-onset Alzheimer’s disease (EOAD) represents the leading cause of degenerative EOD. It is characterized by a relatively high genetic predisposition, with approximately 10% of cases linked to autosomal dominant familial AD caused by mutations in *Presenilin 1 (PSEN1), Presenilin 2 (PSEN2)*, and *Amyloid Precursor Protein (APP)*. Non-amnestic phenotypic variants have also been identified in EOAD, including the logopenic variant of primary progressive aphasia (PPA), posterior cortical atrophy, behavioral/dysexecutive presentations, acalculia, and corticobasal syndrome (CBS) [4].

Frontotemporal dementia (FTD) is the second most common, with the main clinical forms being behavioral variant FTD (bvFTD) and PPA, predominantly in the semantic variant (svPPA) and agrammatic variant (nfPPA) [5]. Patients may develop parkinsonism or motor neuron disease (MND), leading to a broad clinical phenotype that ranges from amyotrophic lateral sclerosis (ALS) to progressive supranuclear palsy (PSP) and CBS [5]. Although most cases of FTD are sporadic, about 20–25% of patients present with a familial form with autosomal dominant inheritance primarily associated with mutations in *C9orf72, Progranulin (GRN),* and *Microtubule-Associated Protein Tau (MAPT)* [6, 7].

Next-generation sequencing (NGS) techniques have enabled the identification of new genes responsible for monogenic dementias and genes associated with phenotypes resembling degenerative dementias [8]. Among these, Colony Stimulating Factor 1 Receptor (*CSF1R*) may play a key role. Several cases of patients clinically diagnosed with FTD, especially in its behavioral variant, have been recently reported to carry a *CSF1R* mutation [9–13]. The *CSF1R* gene consists of 22 exons and encodes for a transmembrane receptor with tyrosine kinase activity expressed in several subpopulations of central nervous system cells such as microglia, while its expression on neuronal progenitor cells (NPCs) is a matter of debate [14, 15]. As a result of interaction with its ligands, *CSF1* and interleukin 34 (IL34), it enables microglia development, self-renewal, differentiation, and survival of NPCs. *CSF1R* also interacts with Triggering Receptor Expressed on Myeloid cells 2 (TREM2) and Immunoreceptor DAP12 (DAP12), regulating the survival of microglia [16].

Variants in *CSF1R* are related to Adult-Onset Leukodystrophy with Axonal Spheroids and Pigmented glia (ALSP), a rare neurological disorder with autosomal dominant transmission and a typical clinical onset in the fourth decade of life [14]. Neuropathologically, it is characterized by white matter degeneration associated with neuronal damage and axonal spurs containing neurofilaments, amyloid and ubiquitin, deformed astrocytes, and pigmented glial cells [14]. ALSP generally presents with a spectrum of neurological symptoms, including cognitive impairment, psychiatric manifestations, and motor dysfunction.

Cognitive disorders frequently manifest as a frontal lobe syndrome characterized by cognitive decline, disinhibition, depression, and poor insight. As disease progresses, a heterogeneous clinical syndrome may develop, which can include aphasia, agraphia, acalculia, pyramidal and bulbar involvement, parkinsonism, cerebellar dysfunction, and seizures [14].

Although no specific therapies are available for *CSF1R*-related leukoencephalopathy (CRL) and many approaches targeting the microglial niche have failed, hematopoietic stem cell transplantation (HSCT) remains the most promising option, with evidence supporting its ability to slow or arrest disease progression [17–19]. Since prompt intervention is crucial for HSCT, it is important to identify suitable candidates at an early stage.

Although several studies have described the clinical and radiological features of CRL, detailed reports integrating clinical, neuroimaging, genetic, and biomarker data remain limited. In this study, we describe the clinical phenotype and genetic findings in four subjects with variants in *CSF1R* referred to an Italian reference center. Further objectives were to identify distinguishing features that may help differentiate CRL from other early-onset neurodegenerative disorders, particularly FTD, and to evaluate the prevalence of CRL in a cohort of cognitively impaired patients from Emilia-Romagna, Northern Italy.

## Methods

### Study population

The cohort analyzed in this study comprised patients with progressive cognitive impairment of suspected neurodegenerative etiology, consecutively referred for genetic analysis between 2005 and 2024 to the Neuropathology Laboratory, IRCCS Institute of Neurological Sciences of Bologna, a reference center for genetic analysis of neurodegenerative diseases. Most samples were referred from the Emilia-Romagna region in Italy. In all cases, the suspicion of a genetic disorder was based on either an early disease onset (before 65 years of age) or a positive family history of neurodegenerative diseases. A total of 2163 subjects were analyzed. A variant in *CSF1R* was identified in 16 samples. Inclusion criteria for the present study were the presence of a variant in *CSF1R* classified as pathogenic, likely pathogenic or hot variant of uncertain significance (VUS) by at least one of the tools used (see below). Exclusion criteria were cognitive deterioration secondary to other causes (e.g., vascular or traumatic forms, etc.) and a biological diagnosis of Alzheimer’s disease (via Amyloid PET or neurodegeneration markers). Four participants were included in this case series. A preliminary description of one participant included in the present study (Patient 2) has already been reported [20]. In the present study, we provide additional clinical, neuroimaging, genetic, and biomarker data that were not available at the time of the original publication, and we contextualize this case within a broader cohort. Clinical and genetic details on patients with *CSF1R* variants not fulfilling previous criteria and therefore being excluded from this study are reported in Supplementary Table 1.

The analysis included a retrospective review of clinical records. The following clinical characteristics were extracted: sex, age at onset, family history of neurodegenerative diseases, past medical history, initial and late symptoms (including language disorders, bulbar signs, behavioral disorders, epilepsy, extrapyramidal symptoms, gait impairment, and motor neuron symptoms).

We also included neuropsychological assessments; however, these were not conducted using a common standardized battery.

### Neuroimaging analyses

Patients underwent neuroimaging analysis using a 1.5 T magnetic resonance imaging (MRI) and 1-mm slice thickness brain CT scan. We assessed cerebral atrophy and white matter abnormalities through visual rating scales [21], abnormal signals in the corpus callosum, dilatation of subarachnoid spaces and of third ventricle, calcifications, diffusion-restricted lesions, gadolinium contrast enhancement, presence of a cavum vergae, and arachnoid cysts. Two neurologists specializing in neuroimaging (AZ and MS) performed the analysis. The review of MRI images was possible for all participants except one (Patient 4).

### Cerebrospinal fluid analyses

CSF samples were obtained by lumbar puncture, centrifuged in case of blood contamination, divided into aliquots, and stored in polypropylene tubes at −80 °C until analysis. CSF t-tau, p-tau181, Aβ42, Aβ40, and neurofilament light chain (NfL) proteins were measured by automated chemiluminescent enzyme immunoassay on the Lumipulse G600II platform (Fujirebio, Gent, Belgium). The mean intra-assay and inter-assay CVs were < 8%. The Aβ42/Aβ40 ratio was calculated as described [22]. Pathological values for the AD core markers were determined according to in-house validated cutoffs. Specifically, a CSF Aβ42/Aβ40 ratio < 0.68 was considered supporting of beta-amyloid deposition, while a CSF p-tau181 > 62 pg/ml was considered indicative of p-tau deposition.

### Genetic analyses

In all participants, *CSF1R* gene analysis was performed through NGS, either dedicated panel or whole-exome sequencing (WES) on DNA extracted from the peripheral blood cells. The sample library was prepared using xGen™ DNA EZ Library Prep Kit (IDT), enriched with xGen Exome Research Panel v2 (IDT) probes, and then sequenced with 2 × 100 bp paired-end reads on a NovaSeq 6000 instrument (Illumina). Sequencing was performed with an average coverage of 131.7X and the coverage 20 × was over 99% of all samples. Bioinformatic analysis followed the GATK v.4.2.0.0 workflow for germline variant discovery, aligning to reference genome GRCh38/hg38. Variants of interest were classified according to the American College of Medical Genetics (ACMG) guidelines [23], and the evaluation of clinical consequences was based on Varsome and or Franklin Genoox genomic data platforms. VUS variants were categorized according to the Association for Clinical Genomic Science (ACGS) best practice guidelines [24]. To obtain an overview of previously reported variants, each one was also checked in ClinVar and in the public version of Human Gene Mutation Database (HGMD) to determine whether it had been previously described. The diagnosis of CRL was made according to criteria established by Konno et al. [25]. **(**Table 1**).**

**Table 1:** Diagnostic criteria for ALSP applied to the included participants

Diagnostic criteria for ALSP [20]	#1	#2	#3	#4
Principal criteria				
Age at onset ≤ 60 years	Y	Y	Y	N
Two or more of the following signs/symptoms:				
Cognitive impairment or psychiatric symptoms	Y	Y	Y	Y
Pyramidal signs	N	Y	N	N
Parkinsonism	N	Y	Y	N
Epilepsy	Y	Y	Y	Y
Autosomal dominant inheritance or sporadic onset	S	S	S	AD
Brain CT/MRI findings				
Bilateral white matter lesions	Y	Y	Y	Y
Callosal thinning	Y	Y	Y	Y
Other causes of leukoencephalopathy, including vascular dementia, multiple sclerosis, and leukodystrophies, can be excluded	Y	Y	Y	Y
Supportive criteria				
Frontal lobe dysfunction based on clinical signs or neuropsychological tests	Y	Y	Y	N
Rapidly progressive course (become bedridden within 5 years after onset)	N	Y	Y	NA
Small calcifications on CT in white matter	Y	Y	Y	NA
Neuropathological findings compatible to ALSP	NA	NA	NA	NA
Exclusionary findings				
Age at onset ≤ 10 years	N	N	N	N
Stroke-like episodes more than twice except for epilepsy	N	N	N	N
Prominent peripheral neuropathy	N	N	N	N
Diagnostic certainty by criteria	DEF	DEF	DEF	DEF

AD, autosomal dominant; DEF, definite; N, absent; NA, data not available; S, sporadic; Y, present

## Results

### Individual description of cases

#### Patient 1

Female, Caucasian, without a family history of neurological disorders and with a history of alcohol abuse. She presented with focal seizures at the age of 48, followed by attentional and memory deficits and a gait disorder. Neurological examination revealed fine postural tremors of the upper limbs bilaterally and a wide-based gait. Neuropsychological evaluation documented multi-domain deficits, including memory and executive functions. CSF analysis showed elevated NfL levels. Brain MRI displayed great global cortical atrophy (GCA 2), predominantly frontal, with an anterior–posterior gradient, along with symmetric periventricular and frontoparietal white matter changes, slightly more pronounced anteriorly, and periventricular frontal calcifications. She was initially diagnosed with epilepsy and major neurocognitive disorder due to toxic-metabolic damage. Genetic analysis identified the c.2381 T > C (p.Ile794Thr) variant in the *CSF1R* gene. It had already been reported in the literature and is listed in ClinVar, where it is classified as pathogenic/likely pathogenic. The prediction tools used in our analysis also support a deleterious effect, reinforcing its potential pathogenicity.

Five years after onset, the patient was not yet bedridden, but exhibited a major neurocognitive disorder. She further experienced seizures, which required adjustment of her anti-seizure medication.

#### Patient 2

Male, Caucasian, without a family history of neurological disorders and no significant clinical history. He developed apathy/abulia and loss of initiative with social withdrawal at the age of 46, followed a year later by obsessive behaviors, craving for sweet foods, global motor and ideational slowing, seizures, and less fluent, more telegraphic speech. Neurological examination was initially unremarkable; however, two years after onset it showed perseveration, motor stereotypies, extrapyramidal signs, and camptocormia. At baseline, neuropsychological evaluation showed a multi-domain mild cognitive impairment (MCI), particularly affecting attentional-executive domains and working memory. CSF analysis revealed elevated NfL levels. Brain MRI showed marked global atrophy, predominantly frontal, with an anterior–posterior gradient. There were also symmetric, periventricular, serpiginous calcifications, and a large arachnoid cyst. Initial diagnosis was bvFTD. Genetic analysis identified the c.2509G > C (p.Asp837His) variant in the *CSF1R* gene. This variant is not reported in international databases and has only been previously described by our group in the same patient. It is classified as likely pathogenic by the tools used. The patient became bedridden and died within  five years of symptom onset.

#### Patient 3

Male, Caucasian, without a family history of neurological disorders and no significant clinical history. He developed multi-domain cognitive deficits, predominantly non-amnesic, associated with apathy and abulia at the age of 51. Within a year, he also developed hyperphagia, speech difficulties, gait apraxia, and generalized seizures. Neurological examination one year after symptom onset revealed spastic hypertonia, perseveration, non-purposeful hand stereotypies, and unstable apraxic gait. Neuropsychological evaluation showed multi-domain cognitive deficits, particularly in language and attentional-executive domains. CSF analysis showed normal chemical–physical properties and markedly elevated NfL levels. Brain MRI showed marked global atrophy, predominantly frontoparietal, with an anterior–posterior gradient and anterior callosal atrophy. There were also symmetric, confluent frontal white matter changes and serpiginous frontal and periventricular calcifications. Initial diagnosis was bvFTD. Genetic analysis identified the c.2517G > C (p.Trp839Cys) variant in the *CSF1R* gene. This variant is not reported in international databases and has not been previously described in the literature, being classified as likely pathogenic by the tools used. Five years after symptom onset, the patient was bedridden.

#### Patient 4

Male, Caucasian, with a paternal history of early-onset cognitive decline and aphasia and no significant clinical history. He presented with seizures at the age of 61, followed within a year by writing difficulties and word-finding issues. Neurological examination revealed motor impersistence. Neuropsychological testing showed deficient phonemic fluency and borderline performance in executive functions. CSF analysis showed increased NfL and t-tau levels. Brain MRI (report only, images not reviewed) revealed severe atrophy of the left frontal lobe, particularly involving the superior and middle frontal gyri, with consequent widening of sulcal CSF spaces and ex-vacuo dilatation of the frontal horn of the left lateral ventricle. There was reduced representation of lobar white matter with subcortical gliotic hyperintensities and atrophy of the anterior third of the corpus callosum. Initial diagnosis was epilepsy and suspected cognitive impairment. Genetic analysis identified the c.2649_2651delGAA (p.Lys883del) variant in the *CSF1R* gene. This variant is not reported in international databases and has not been previously described in the literature, being classified as VUS by the tools used.

### Clinical findings

A total of four patients (3 males, 1 female) were included in the case series. Detailed clinical presentations of these patients are summarized in Table 2. The mean age at onset was 51.5 years (range 46–61 years). A single patient (25%) had a positive family history of early-onset cognitive decline.

**Table 2:** Clinical features of the included participants

	#1	#2	#3	#4
Language disorders				
Non-fluent aphasia	A	LO	LO	LO
Logopenic aphasia	A	A	A	A
Semantic aphasia	A	A	A	A
Bulbar signs				
Dysarthria	A	A	A	A
Dysphagia	A	A	A	A
Behavioral disorder				
Disinhibition	A	A	A	A
Apathy or inertia	A	PO	PO	A
Loss of sympathy or empathy	A	A	A	A
Neuropathological findings compatible to ALSP	NA	NA	NA	NA
Perseverative, stereotyped, or compulsive behaviors	A	LO	A	A
Hyperorality or dietary changes	A	LO	LO	A
Depression or emotional instability	A	A	A	A
Epilepsy	PO	LO	LO	PO
Extrapyramidal symptoms/signs	A	LO	LO	A
Gait impairment	PO	LO	LO	A
Motor neuron symptoms/signs				
First motor neuron	A	A	LO	A
Second motor neuron	A	A	A	A
Sleep disorders	A	LO	A	A
Symptoms progression during the first year	Y	Y	Y	Y
Diagnostic criteria for bvFTD	NM	PR	PR	NM

A, absent; LO, present after the first year; NA, data not available; NM, not met; PO, present at onset; PR, probable; Y, present

Behavioral disturbances, predominantly apathy, were reported as initial symptoms in two patients (50%). In both, hyperorality and compulsive behaviors developed after the first disease year. Epilepsy was observed at onset in two (50%) and occurred in all patients (100%) with disease progression. Variable gait disturbances were the initial symptom in one patient (25%) and were observed in three (75%) at a later stage of the disease. Language disturbances, mainly non-fluent aphasia, developed in three patients (75%) throughout the disease. Extrapyramidal symptoms, mainly parkinsonism, developed in two (50%) during the clinical course. One out of four patients (25%) developed signs of motor neuron involvement after the first year of disease.

Follow-up data at  five years were not available for one patient; two patients (50%) were bedridden within  five years of onset, and one patient (25%) died within the same timeframe.

### Neuropsychological findings

Table 3 summarizes the results of the cognitive assessments. Unfortunately, the neuropsychological testing varied, as did the assessment time after the onset of symptoms (ranging from 6 months to 4 years) and follow-up data were not available for all patients. Despite these limitations, language tests (phonemic and semantic fluency) were impaired in 2/4 patients (50%), while one patient scored at the lower limits. Memory, assessed using the RAVLT and FCRST tests, was impaired in 2/4 patients (50%). The Stroop test, which assesses frontal and executive functions, yielded pathological scores in 3/4 patients (75%). Three patients (75%, patients 1, 2, and 3) had longitudinal neuropsychological assessment results available, showing cognitive deterioration in two of them (patients 2 and 3).

**Table 3:** Cognitive assessment results in the included participants

	#1	#2	#3	#4
Assessment time from symptoms onset	4 yrs	1 yrs	6 months	1 yrs
Global cognition (Mini Mental State Examination)	26/30	27/30	28/30	30/30
Episodic memory (Rey auditory verbal learning test and/or Free and Cued Selective Reminding Test)	A	N	A	N
Working memory (digit span forward and backward)	A	A	N	NA
Attention (Stroop Test and/or Multiple Features Target Cancelation Test	A	A	A	N
Executive functions (Frontal Assessment Battery and/or Stroop Test, Raven’s Progressive Matrices, Wisconsin Card Sorting Test)	A	A	A	N
Visuospatial ability (Rey-Osterrieth Complex Figure and/or drawing tests)	A	N	A	N
Language (Phonologic verbal and Semantic Fluency Tests)	N (lower limit)	N	A (both SE and PH)	A (PH), N (SE)

A, altered; N, normal; NA, data not available; PH: phonological verbal fluency; SE: semantic fluency

### Neuroradiological findings

**Fig. 1:**
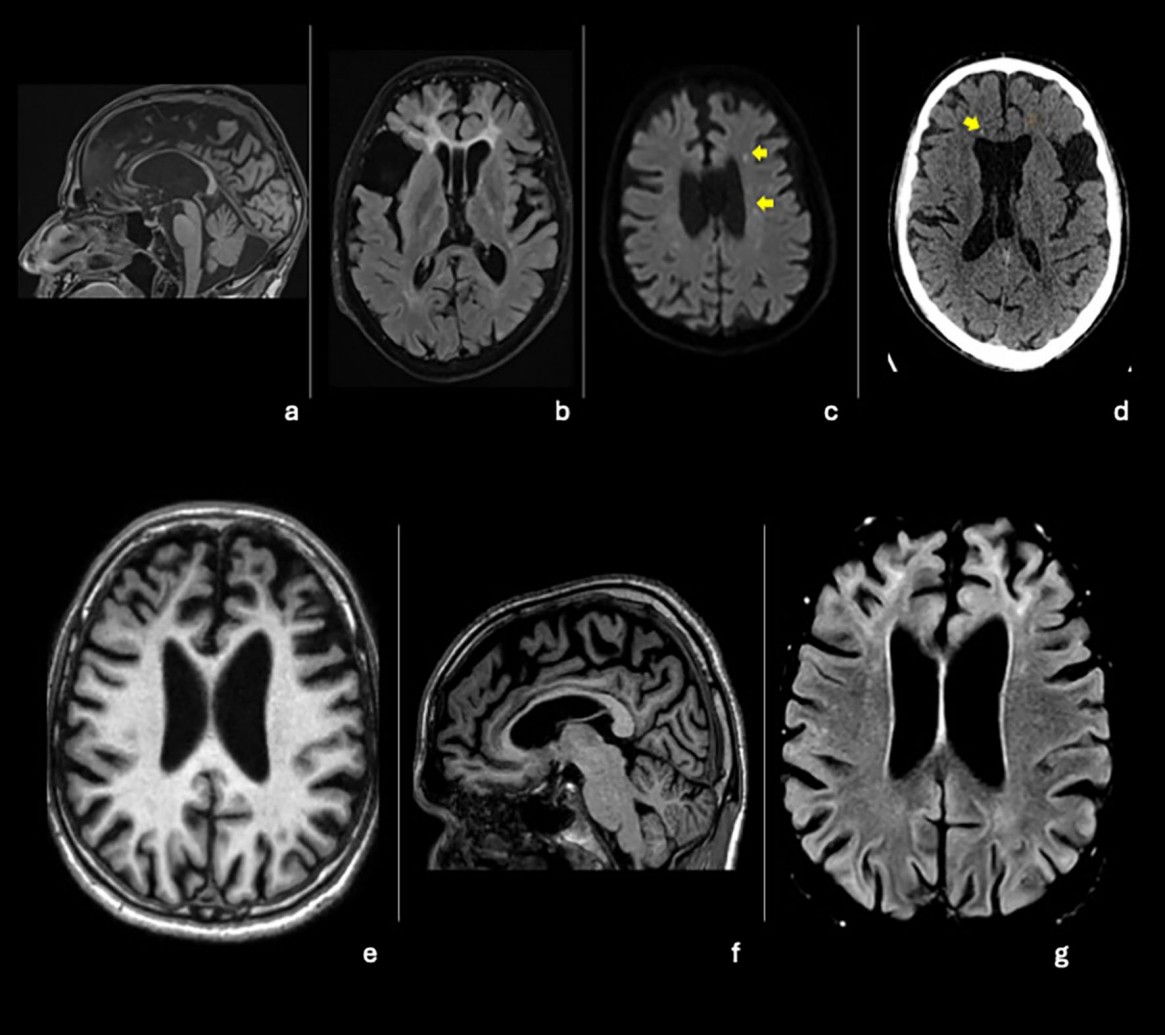
Imaging abnormalities in selected included participants. Brain T2-FLAIR MRI sequence shows corpus callosum atrophy, frontal hyperintensities, cavum vergae, and an arachnoid cyst in patient 2 (**a, b**). Diffusion-weighted MRI and thin-slice CT (1 mm) (**c, d**) show the presence of calcifications in patient 2 (yellow arrows). Brain T1-MRI shows frontal atrophy in patient 3 (**e**); T2-FLAIR MRI sequences reveal thinning of the corpus callosum and frontal hyperintensity in patient 3 (**f, g**)

The radiological data of three patients were directly assessed, while for one patient (patient 4), only the report was available. Neuroradiological findings are summarized in Table 4. Medial temporal atrophy (MTA ≥ 2) was found in 2/3 (66.7%) subjects; predominant frontal atrophy exceeding the degree expected for age (GCA-F scores of 2 and 3) was observed in 4/4 (100%), and anterior–posterior atrophy gradient was found in 3/3 (100%). Callosal atrophy, mainly anterior, was found in 4/4 (100%) patients. Symmetric white matter involvement, predominantly in the frontal and periventricular regions, was present in 4/4 (100%) subjects. Calcifications, identified in SWI and GRE sequences, with predominantly serpiginous pattern were observed in 3/3 (100%) examined patients. Additional findings included the presence of a cavum vergae and an arachnoid cyst in one patient (Fig. 1).

**Table 4:** Neuroradiological findings in the included participants

	#1	#2	#3	#4
Global cerebral atrophy (GCA)	2	3	3	NA
Medial temporal lobe atrophy (MTA)	2	2	1	NA
Parietal lobe atrophy (Koedam)	1	2	3	NA
Frontal atrophy (GCA-F)	2	3	3	3
Anterior–posterior gradient	Y	Y	Y	NA
Callosal atrophy				
Presence	Y	Y	Y	Y
Location	Anterior	Anteromedial	Anterior	Anterior
Disproportionately Enlarged Subarachnoid Space Hydrocephalus (DESH)	Y	N	N	Y
Third ventricle (millimeters)	11.5	13.7	7	NA
White matter changes				
Presence	Y	Y	Y	Y
Confluent areas	Y	Y	Y	(not further described)
Location	F, P, PV	F	F	
Symmetry	Y	Y	Y	
Calcifications				
Presence	Y	Y	Y	NA
Pattern	-	Ser	Ser	
Location	F, PV	F, PV	F, PV	
Diffusion-weighted imaging changes	N	Y	N	N
Gadolinium contrast enhancement	N	N	Np	NA
Cavum vergae	N	Y	N	N
Arachnoid cysts	N	Y	N	N

F, frontal; N, absent; NA, data not available; Np, not performed; P, parietal; PV, periventricular; Ser, serpentine; Y, present

### Cerebrospinal fluid and genetic findings

In all subjects included, NfL levels were elevated. Values above 10,000 pg/ml, suggesting massive neurodegeneration, were reported in 1/4 (25%).

In none, the CSF profile was suggestive of Alzheimer’s disease neuropathological changes. Notably, two participants (patients 2 and 4) showed high CSF t-tau levels in the absence of Aβ or tau deposition. CSF findings are summarized in Table 5.

**Table 5:** Cerebrospinal fluid findings in the included participants

	#1	#2	#3	#4
t-tau protein (v.n 90–450 pg/ml)	N 183 pg/ml	A 509 pg/ml	N 264 pg/ml	A 1330 pg/ml
p-tau_181_ protein (v.n 15–62 pg/ml)	N 16 pg/ml	N 39 pg/ml	N 28 pg/ml	N 25 pg/ml
Amyloid Beta 1–42/1–40 ratio (n.v. 0.68–1.7)	N 1.06	N 0.88	N 0.99	N 1.14
Neurofilament light chain (n.v. 340–650 pg/ml)	A 1916 pg/ml	A 10,400 pg/ml	A 4470 pg/ml	A 2774 pg/ml

A, out of range; N, in range; p-tau181, phosphorylated tau protein at residue 181; t-tau, total tau protein

Genetic findings in the selected subjects are summarized in Table 6. Only the variant of Patient 1 had already been reported. The variants found in patients 1, 2, and 3 were classified as pathogenic/likely pathogenic by both prediction tools, whereas that detected in Patient 4 was considered as VUS.

**Table 6:** Genetic findings in the included participants

	#1	#2	#3	#4
Nucleotide change	c.2381 T > C	c.2509G > C	c.2517G > C	c.2649_2651 del
Protein change	p.Ile794Thr	p.Asp837His	p. Trp839Cys	p. Lys883del
Franklin ACMG Classification	P (PS4, PM1, PM2, PP3, PM5)	LP (PM2, PP3, PM1, PM5)	LP (PM2, PP3, PM1)	wVUS (PM2, PM4)
VarSome Classification	P (PS3, PM1, PM2, PP3, PM5)	LP (PP3, PM1, PM5, PM2)	P (PS1, PP3, PM1, PM2)	hVUS (PM1, PM4, PM2)

hVUS, hot variant of unclear significance; LP, likely pathogenic; P, pathogenic; wVUS, warm variant of unclear significance

Population allele frequencies referred to the European (non-Finnish) population reported on GnomAD Exomes v24.0 expressed as allele count/allele number

## Discussion

We identified four cases of *CSF1R*-related disease in a cohort of unrelated individuals from northern Italy, presenting clinically with a FTD like disorder, collected over a 19-year period.

The prevalence of the disease observed in our cohort (4/2163) is substantially higher than that recently reported in a similar study from Wade et al. (~ 1/3500) [26]. However, this comparison must be interpreted in light of the different methodological approaches of the two studies. Wade et al., using a genome-first approach, analyzed a large, unselected population from the UK Biobank [26]. As acknowledged by the authors themselves, this sampling strategy may lead to an underestimation of the actual prevalence of pathogenic *CSF1R* variants. In our study, by contrast, the cohort was selected based on the presence of early-onset cognitive decline, introducing an ascertainment bias in the opposite direction. Specifically, enrichment for phenotypes compatible with CRL increases the likelihood of identifying pathogenic variants compared to an unselected population. Therefore, the high prevalence observed in our study overestimates the actual prevalence of *CSF1R* pathogenic variants in the general population.

CRL is an autosomal dominant disorder; however, in our cohort, a single participant (Patient 4) had a family history of cognitive decline. The frequent sporadic cases reported in the literature, although partly explained by de novo mutations, may also result from incomplete penetrance or mosaicism [27–30]. It is also possible that a “second hit” is required for disease manifestation, such as environmental factors (e.g., glucocorticoid exposure, head trauma or viral infection) or the presence of additional unidentified genetic variants in cis or in trans around the *CSF1R* locus. These mechanisms may explain why not every variant carrier develops the disease [26, 29]. The onset of CRL can occur from early adulthood to the eight decade of life. In our cohort, the mean age at onset was 51 years, approximately eight years later than that reported in the literature [14, 27, 31], yet still slightly earlier than that observed in more common dementing disorders [32–34].

Patients with CRL present a wide spectrum of neuropsychiatric symptoms in adulthood. Neuropsychological data from our case series showed that 75% of patients exhibited impairment in frontal and executive functions, detected through Digit Span and Stroop tests. However, unlike what was reported by Rush et al., our cohort also exhibited alterations in memory and language domains [35]. This discrepancy may reflect greater clinical heterogeneity or differences in disease stage at the time of assessment.

CRL is classically described with phenotypes resembling FTD, parkinsonian syndromes, and progressive multiple sclerosis-like presentations [14, 28]. In our series, behavioral disturbances were the initial manifestation in Patients 2 and 3, resulting in a clinical presentation initially indistinguishable from bvFTD. Clinical assessment and neuropsychological testing alone, therefore, make the differential diagnosis challenging. Some red flags, however, seem to emerge from this case series and may help orienting toward CRL: a younger age at onset, a faster progression compared with FTD, and the presence of seizures, which are generally rare in FTD [36].

Neuroimaging, specifically progressive white matter lesions, brain atrophy, and calcifications, also plays a central role in the diagnosis of CRL and provides valuable support in the differential diagnosis [37]. Although white matter abnormalities may also occur in FTD, particularly in cases with *GRN* mutations, they demonstrated a more distinctive pattern in our cohort. This includes marked frontal involvement, both subcortical and juxtacortical, along with involvement of commissural fibers [38]. Moreover, pronounced thinning of the corpus callosum, especially at early stages [27], and calcifications represent additional distinguishing features. In our cohort, these were detected in all patients, confirming their high diagnostic value.

The role of CSF biomarkers, especially NfL protein, in the diagnostic approach to patients with CRL deserves a final comment. Neurofilament light chain is emerging as a reliable biomarker of subcortical damage in various neurological disorders and has also proven to be a useful tool in the diagnostic approach to patients with neurodegenerative diseases and cognitive impairment [39–41] Recently, markedly elevated NfL levels in both CSF and plasma have been reported in individuals with CRL, including pre-symptomatic carriers of pathogenic variants in the *CSF1R* gene [42]. In our cohort, we confirm a significant elevation of CSF NfL in the majority of patients, with one individual showing levels exceeding 10,000 pg/ml. The two participants presenting with a bvFTD-like phenotype (Patient 2 and 3) showed CSF NfL levels that exceeded those usually associated with this clinical syndrome.

Adopting a consistent diagnostic approach in patients with EOD and ensuring the early identification of those suspected of having CRL can greatly facilitate their timely referral to specialist care and increase the likelihood of an accurate diagnosis. This is particularly relevant in light of the emerging therapeutic strategies currently under investigation for CRL.

Although the clinical trial with TREM2 agonist monoclonal antibodies (NCT05677659) was discontinued due to lack of efficacy, data collected from patients undergoing HSCT have shown promising results [18, 43]. HSCT appears to halt disease progression after the first 6 months post-transplant, typically characterized by an initial clinical and radiological worsening, as demonstrated by the arrest of cerebral demyelination, the marked reduction in plasma/serum NfL levels, and the frequent stabilization or even improvement of clinical symptoms. These findings support HSCT as an important therapeutic option, suggesting that its disease-modifying effects may continue for years after transplantation and are likely more pronounced when treatment is initiated early, while it is not recommended for asymptomatic carriers given the incomplete penetrance of the disease [18].

A major strength of this study is that all included patients had a genetically confirmed diagnosis of CRL. Although CRL is one of the most prevalent leukodystrophies, it remains underdiagnosed. Our cohort of four patients, characterized through detailed clinical, radiological, genetic, and CSF biomarker assessments, provides useful additional information to the existing literature.

However, this study is subject to limitations. Data collection was retrospective, with patients assessed at different stages of the disease, and follow-up data were not consistently available for at least five years in some cases. Moreover, uniform neuropsychological assessments were not performed for all patients, and direct access to neuroimaging was not obtained for one patient. Therefore, these findings should be interpreted with caution and require validation in larger, prospective cohorts.

In conclusion, we outlined the clinical, neuroimaging, and genetic features of four patients with CRL and identified key elements that may help distinguish it from other early-onset neurodegenerative disorders, particularly FTD. Given the high frequency of de novo variants and their incomplete penetrance, genetic testing should be pursued even in patients without a positive family history. Overall, these findings may contribute to improve the diagnostic accuracy and clinical management in CRL.

## Supplementary Information

Below is the link to the electronic supplementary material.

Supplementary file1 (DOCX 18 KB)

## Data Availability

Pseudonymized data supporting the findings of the present study are available upon reasonable request to the corresponding author.
