# Supplementary material for: CSF1R mutations in an Italian population of early-onset dementia: a case series
Source: J Neurol. 2026 Feb 4;273(2):115. doi: 10.1007/s00415-026-13643-1 (PMC12868039; doi:10.1007/s00415-026-13643-1)
Supplement: Supplementary file 1 — Supplementary file1 (DOCX 18 KB) [file 415_2026_13643_MOESM1_ESM.docx]

**Supplementary Table 1 – Genetic and clinical characteristics of participants with variants in *CSF1R* gene excluded from the study**

|  | **Variant** | **Franklin ACMG Classification** | **Varsome Classification** | **ClinVar** | **Clinical details** |
| --- | --- | --- | --- | --- | --- |
| **#1** | c.306A>G  p.Lys102= | icVUS  (PM2,BP4) | icVUS  (PM2,BP4) | NA | DO at 65, rapidly progressive cognitive impairment. Neuropathological diagnosis of AD. |
| **#2** | c.1626+3G>A | B (BS1,BS2,BP4,BP6) | B (BS1,BS2,BP4,BP6) | B/LB | DO at 65, rapidly progressive cognitive impairment associated with cerebellar and extrapyramidal signs. CSF biomarkers not suggestive of AD. |
| **#3** | c.2850C>A  p.His950Gln | icVUS  (PM2,BP4) | LB  (BP4,PM2) | NA | DO at 62, slowly progressive language disorder with late multidomain involvement. CSF biomarkers suggestive of AD. |
| **#4** | c.1477A>G  p.Ser493Gly | icVUS  (PM2,BP4) | LB (BP4,PM2) | NA | DO at 60, slowly progressive memory disorder with behavioural symptoms. CSF biomarkers suggestive of AD |
| **#5** | c.1133G>A  p.Arg378His | icVUS  (PM2,BP4) | LB  (BP4,PM2) | VUS | Subject with a central hypersomnia syndrome. Onset of cognitive impairment at 58. Results of CSF biomarkers not available. |
| **#6** | c.2239G>A  p.Gly747Arg | B  (BS1,BS2,BP4,BP6) | B  (BP6, BS1,BS2,BP4,PM1) | B/LB | DO at 59, slowly progressive language and memory impairment. CSF biomarkers suggestive of AD. |
| **#7** | c.99C>G  p.Lys33Asn | icVUS  (PM2,BP4) | LB  (BP4,PM2) | NA | DO at 55, slowly progressive language and behavioural impairment. CSF biomarkers not suggestive of AD. |
| **#8** | c.1810C>A  p.Leu604Met | cVUS  (PM2) | colVUS  (PM1,PM2,BP4) | NA | DO at 63, slowly progressive executive and visuospatial impairment. Clinical diagnosis of DLB. CSF a-syn SAA positive. |
| **#9** | c.2227G>T  p.Asp743Tyr | cVUS  (PM2) | icVUS  (PM1,PM2,BP4) | NA | DO at 46, slowly progressive language and memory impairment. Results of CSF biomarkers not available |
| **#10** | c.641G>A  p.Arg214Gln | LB  (BP4,BP6,PM2) | LB (BP4,BP6,PM2) | LB | DO at 64, slowly progressive parkinsonian syndrome with cognitive impairment. Results of CSF biomarkers not available. |
| **#11** | c.2215G>A  p.Glu739Lys | cVUS  (PM2) | LB  (BP4,PM1,PM2) | VUS | DO at 62, slowly progressive language disorder with limb dystonia and parkinsonian syndrome. CSF biomarkers not suggestive of AD. |
| **#12** | c.722-724del  p.Asn241del | wVUS  (PM2,PM4) | icVUS  (PM2,BP4) | NA | DO at 63, rapidly progressive pyramidal syndrome with bulbar impairment. Clinical diagnosis of motor neuron disease. Markedly elevated CSF neurofilament light chain protein. |

Abbreviations: AD, Alzheimer’s disease; a-syn, alpha-synuclein; B, benign; CSF, cerebrospinal fluid; cVUS, cool Variant of uncertain significance; colVUS, cold Variant of uncertain significance; DO, disease onset; icVUS, ice cold Variant of Uncertain Significance; LB, likely benign; NA, not available; SAA, seed amplification assay; VUS, Variant of uncertain significance; wVUS, warm Variant of uncertain significance.
